# Study of DNA adsorption on mica surfaces using a surface force apparatus

**DOI:** 10.1038/srep08442

**Published:** 2015-02-13

**Authors:** Yajing Kan, Qiyan Tan, Gensheng Wu, Wei Si, Yunfei Chen

**Affiliations:** 1 Jiangsu Key Laboratory for Design and Manufacture of Micro-Nano Biomedical Instruments, and School of Mechanical Engineering, Southeast University, Nanjing 211189, China

## Abstract

We report our studies on the adsorption properties of double-stranded DNA molecules on mica surfaces in a confined environment using a surface force apparatus. Specifically, we studied the influence of cation species and concentrations on DNA adsorption properties. Our results indicated that divalent cations (Mg^2+^ and Co^2+^) preferred to form uniform and moderately dense DNA layers on a mica substrate. By measuring the interactions between DNA-coated mica and bare mica in an aqueous solution, obvious adhesion was observed in a cobalt chloride solution, possibly due to the ion-correlation attraction between negatively charged DNA and the mica surface. Furthermore, the interaction differences that were observed with MgCl_2_ and CoCl_2_ solutions reveal that the specific adsorption behaviors of DNA molecules on a mica substrate were mediated by these two salts. Our results are helpful to elucidate the dynamics of DNA binding on a solid substrate.

The adsorption kinetics of DNA on various solid substrates have been a popular research topic in previous decades due to their many applications, particularly in the development of DNA-based devices, such as biosensors^1^,^2^,^3^, micro arrays^4^,^5^ and transistors^6^,^7^. The atomically smooth surface and stable chemical properties of Muscovite mica make it an ideal substrate for DNA adsorption, and it has been commonly used in experimental research, such as studies of DNA conformation and DNA interactions with solid substrates. However, a DNA molecule is typically negatively charged due to its sugar phosphate backbone, which results in a loose bind with negatively charged mica surfaces in most aqueous environments thanks to electrostatic repulsion. To achieve tight adsorption of DNA on a mica surface, the mica surface is commonly pretreated with divalent cations^8^,^9^. Alternatively, physical or chemical modification of a mica surface is another effective approach to anchor DNA. For example, lipid bilayers containing nucleic acid bases can be deposited on a mica surface via a Langmuir-Blodgett film, which is very useful in the direct measurement of the interaction between DNA bases^10^. A mica surface can also be functionalized, such as with aminosilanes, to construct a positively charged surface to adsorb DNA^8^.

Among these methods, immobilization of DNA on mica or other minerals by adding divalent or multivalent cations is relatively simple and practical but has an unclear binding mechanism. The adsorption of polyelectrolytes on a like-charged solid surface, which is a counterintuitive phenomenon, was thought to be dominated by the ion-correlation effect^11^ or typical electrostatic attraction mediated by divalent cations^12^. In the presence of certain divalent cations, the surface charge can be physically^11^ or chemically neutralized^12^ or even inverted from negative to positive in advance of DNA adsorption. Transitional metals, such as Ni^2+^, Co^2+^ or Zn^2+^, are preferable species for tight DNA binding compared to other divalent cations, such as Mg^2+^ or Ca^2+^, even though they all have similar radiuses^9^. In addition to cation species, the DNA adsorption and corresponding conformation are also influenced significantly by pH^13^,^14^, ionic strength^14^,^15^, DNA conformation in solutions^13^,^16^ and competition between monovalent and divalent cations^11^,^15^,^17^. Despite the breadth of prior research, the ion-mediated binding mechanism and its influence on DNA adsorption kinetics have not yet been clearly understood.

The detection of DNA adsorption on a solid substrate is commonly performed by a number of techniques including atomic force microscopy (AFM)^8^,^18^, quartz crystal microbalance^19^,^20^, ellipsometry^21^,^22^, and X-ray photoelectron spectroscopy (XPS)^23^. In this study, we used a surface force apparatus (SFA), which has a distance resolution of 0.1 nm and a force sensitivity of 10 nN. After 40 years of development, SFA has been widely used in the study of intermolecular and interface interactions^24^. Compared with AFM, SFA is better for measuring the interaction between two surfaces in a wet environment to study the influence of solution conditions. In addition, this device can be used to effectively characterize the adsorption of biological materials on solid substrates, such as the adsorbed film thickness, refractive index and adsorption density^25^,^26^,^27^.

In this work, we used the SFA technique to study the adsorption properties of double-stranded *λ* -DNA on a mica substrate. Specifically, we investigated the influences of salt species and concentrations on DNA adsorption. Additionally, we measured the interactions between DNA-coated mica and bare mica surfaces in different buffer solutions. An attractive interaction between the DNA layer and mica was observed only when the mica surface was pretreated with cobalt salt. It was also found that the attraction magnitude could be enhanced by increasing the ionic strength of divalent cations in the bulk solution. This adhesion was likely induced by the ion-correlation attraction between the phosphate backbone of DNA molecules and the mica surface, which dominates DNA adsorption on mica.

## Methods

### Chemicals

*λ*-DNA (48502 bp) was purchased from TAKARA BIO Inc. (Chengdu, China). HEPES, sodium chloride, magnesium chloride, and cobalt chloride were purchased from Sigma-Aldrich. Buffer solution was prepared with 10 mM HEPES buffer and 10 mM of one of the following chemicals: NaCl, MgCl_2_, and CoCl_2_. The resulting solution was titrated to pH 7.5. As all buffers prepared contained the same 10 mM HEPES component, the buffer solutions discussed below will be referred to by the cation species. Deionized water was used in all glassware cleaning and solution preparation. All the buffer solutions were filtered using syringe filters with a 0.22 μm pore size (Whatman).

### Surface preparation

The stock of 300 μg/ml *λ* -DNA solution (10 mM Tris-HCl, 1 mM EDTA, pH 8.0) was diluted to 50 μg/ml with one of the above buffer solutions at pH 7.5. As the substrate for DNA adsorption, a freshly cleaved mica surface was rinsed with the same buffer solution used in DNA dilution. Then, 20 *μ*l of diluted DNA solution was deposited on the rinsed mica surface for DNA adsorption. After a 20-minute incubation, the surface was flushed thoroughly with an excess of buffer solution to remove unbound DNA molecules, and a droplet of buffer solution was added to the surface. The DNA-treated surface was stored in a petri dish for further use.

### Surface force apparatus

Adsorption properties of *λ* -DNA on mica substrate as well as the force-distance profiles between the adsorbed DNA layer and mica surface were studied using a surface force apparatus, SFA 2000 (SurForce LLC, Santa Barbara). More details on SFA 2000 can be found in previous studies^24^. Here, a DNA-coated mica surface was mounted into the SFA chamber, facing an untreated mica surface in a cross-cylinder geometry. The droplet left on the DNA-coated mica bridged the two surfaces, providing a buffer environment. During each force-run, the interaction between two surfaces was obtained as a function of distance *D* when two surfaces were driven into contact and then separated from each other. In each approaching process, the surfaces are visualized optically with multiple beam interferometry (MBI) using “fringes of equal chromatic order” (FECO). From the positions of the colored FECO fringes observed in the spectrogram, the distance *D* between the two atomically smooth mica surfaces can be measured at the angstrom resolution level, while *D* = 0 denotes the two bare mica surfaces contact in air. To study the influences of cations, the droplet between two mica surfaces could be replaced by simply flushing the surface gap with an excess of buffer solution. The experiments described here were reproducibly repeated at least two times. The adhesive interaction energy per unit area between two flat surfaces can be obtained according to Johnson-Kendall-Roberts theory (*E*_ad_ = *F*_ad_/1.5π*R*) for elastic adhesive contact, where *F*_ad_ is the measured adhesion force and *R* is the radius of the surface^28^. The refractive index of the medium confined between two mica surfaces was determined with the reported method^24^.

## Results and discussion

### Effect of cation valance and concentration on DNA adsorption on mica surfaces

**Figure 1:**
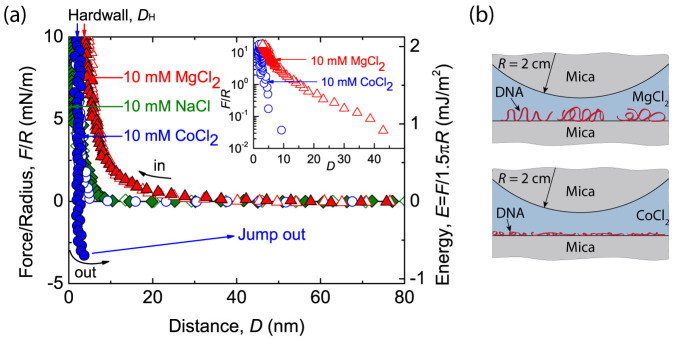
(a) Force-distance profiles between the DNA layer and mica when the DNA is initially incubated on mica in a buffer solution containing 10 mM of MgCl_2_ (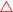 and 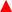), CoCl_2_ (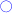 and 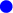) or NaCl (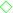 and 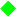). Open symbols(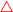, 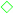 and 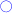): the force curves when two surfaces are brought together (in-run); solid symbols (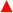, 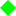 and 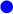): the force curves when two surface are separated (out-run). The semi-log plots of the in-run profiles in both MgCl_2_ and CoCl_2_ are shown in the inset. (b) Schematics of the DNA conformation adsorbed on the mica substrate in the SFA incubated in MgCl_2_ or CoCl_2_.

To investigate the effect of cation species on DNA adsorption, the DNA molecules were incubated on mica in a buffer solution containing monovalent cations (NaCl) or divalent cations (MgCl_2_ or CoCl_2_). The normal force-distance profiles measured from SFA were shown in Figure 1(a). Once two surfaces are brought into contact, steric repulsion inhibits further surface approach under applied compression, which appears as a vertical area in the force curve at a small distance. This distance *D*_H_ is referred to as the hardwall, which may represent the thickness of the DNA layer confined between the two mica surfaces at contact. In cases where the buffers contained 10 mM of divalent metal ions, the hardwall was 2.8 ± 0.2 nm for MgCl_2_ and 2.2 ± 0.1 nm for CoCl_2_. Given that the diameter of a double-stranded DNA (dsDNA) molecule is approximately 2.2 nm^29^, the measured thickness of the DNA layer on mica substrate in each solution indicated the adsorption of the mono *λ* -DNA layer. We also consider this adsorption to be tight enough for SFA studies because the force curves were reproducible when the DNA layer was under a high compression load of over 10 mM/m at the surface contact.

In comparison, when the DNA molecules were initially incubated on mica in 10 mM NaCl solution, the measured hardwall was less than 1 nm. The interaction in both NaCl and MgCl_2_ solutions were similar to measurements in DNA-free control experiments (Figure S1 and S2). It is believed that the DNA molecules failed to adsorb on the mica surface in 10 mM NaCl solution due to electrical double layer (EDL) repulsion and thermal fluctuation. Although monovalent cations, as reported by others^13^,^30^, can be utilized to adsorb DNA, they exhibit very low efficiency compared to divalent cations. Furthermore, because HEPES has the potential to enhance DNA binding to mica due to its two positive charges^8^, our results in NaCl solution demonstrate that HEPES molecules in buffer are unable to help the DNA adsorb on the mica surface.

Generally, it is believed that the counterion correlation is the main cause of DNA adsorption on a mica surface^11^. Therefore, it is expected that the DNA molecules may adsorb on a mica surface immersed in multivalent salt solutions with the help of multivalent cation correlations. This can explain why the DNA molecules cannot adsorb on mica surfaces in NaCl solution. An interesting finding in our measurements is that the DNA molecules have different conformations while they are adsorbed on a mica surface immersed in MgCl_2_ or CoCl_2_ solutions. A long-range repulsion starting at a distance of approximately 40 nm in MgCl_2_ solution was detected when the two mica surfaces were driven to approach each other, whereas no such obvious repulsion was measured in CoCl_2_ solution until contact. A semi-log plot of the force-distance profile during surface approach (Figure 1(a) inset) shows the long-range repulsion observed in MgCl_2_ solution has an exponential decay length of approximately 20 nm, which is much longer than that of the EDL Debye length at 10 mM divalent salt^31^, and implicates the steric effect induced by the adsorbed DNA molecules. Considering that the repulsion range is close to the known persistence length of a dsDNA molecule (approximately 50 nm^32^) and the existence of Mg^2+^ might introduce DNA bending and subsequently reduce the actual persistence length^33^, we propose that the DNA molecule in MgCl_2_ behaves as a condensed wormlike coil with trains attached to the mica, while the other segments form free loops or tails in the solution, as illustrated in Figure 1(b). However, no long-range steric or other repulsion was measured in CoCl_2_ solution, indicating that the DNA molecules were lying flat on the mica substrate in a two dimensional configuration. In a previous X-ray standing wave study of the divalent cation mediated adsorption of single-stranded polynucleotides to a negatively charged silica surface, the transition metal Zn^2+^ ions were directly observed to be sandwiched between the adsorbed layer and the substrate^34^. Moreover, it has been known that transition metals are able to bind to base oxygen and nitrogen in the DNA groove as well as to the phosphate backbone^35^,^36^, which enhances the DNA binding on the mica surface and therefore may condense the DNA structure to a two dimensional surface during adsorption. Ion-mediated DNA condensation in bulk solution normally requires a cation valance of equal to or greater than 3^37^,^38^, but our result exhibited a high likelihood of condensed DNA molecules adsorbing on a solid surface in the presence of divalent cations, which is consistent with previous X-ray diffraction studies^39^. In addition to the difference in repulsion, the adhesion forces between the DNA-adsorbed mica and the bare mica surfaces also demonstrate the difference between CoCl_2_ and MgCl_2_ solutions. The adhesion force could only be measured when the DNA was deposited on the mica surface in CoCl_2_ solution, while no attractive force was measured in MgCl_2_.

The refractive indices of the medium between two mica surfaces were obtained when the two surfaces were in contact, which could demonstrate the adsorbed amount of DNA on the mica. The average refractive indices are 1.404 ± 0.003 for MgCl_2_ and 1.416 ± 0.007 for CoCl_2_. Given the refractive index of 1.333 for water and 1.53 for bulk DNA, the corresponding volume fraction of the adsorbed DNA layer can be determined by Equation (1):
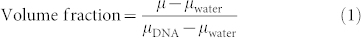
where *μ* is the refractive index of the medium confined between two micas, *μ*_water_ is the refractive index of water, and *μ*_DNA_ is the refractive index of bulk DNA.

The volume fraction or adsorption densities of DNA molecules adsorbed on a substrate are 36.8% ± 2% for MgCl_2_ and 42% ± 4% for CoCl_2_, indicating that a moderately dense DNA layer was adsorbed on the mica substrate in each case. The reduced adsorption in MgCl_2_ compared to that in CoCl_2_ was likely due to the relatively more condensed conformation of DNA molecules. To form a more dense monolayer, the deposited solution with higher DNA concentration may be used in future studies^16^.

Once the volume fraction of DNA on mica is obtained, we can estimate the DNA adsorption amount. For instance, in the CoCl_2_ case, the thickness of the DNA layer is 2.23 nm, and the coverage area of a mica surface on a silicon disk is approximately 0.5 cm^2^. Assuming *λ* -DNA has a density of 1.7 g/cm^3^^40^, the adsorption mass is calculated to be 8.0 × 10^−8^ g, which is only 8% of the original DNA mass used for deposition.

### Desorption of DNA from mica surfaces with highly concentrated monovalent cations

**Figure 2:**
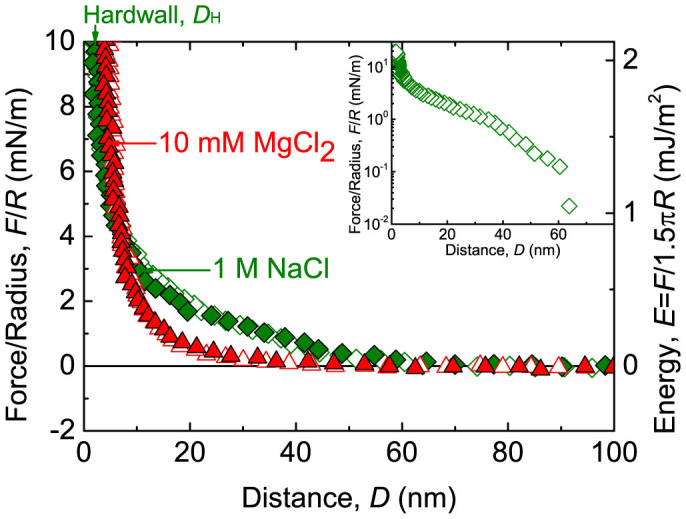
The desorption of the DNA layer on a mica substrate induced by the competition between monovalent and divalent cations. After DNA adsorption in 10 mM MgCl_2_ (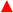), the buffer was replaced by NaCl solutions with concentrations of 10 mM, 100 mM and 1 M (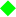). The force curves for 10 mM and 100 mM NaCl are not shown. Inset shows the semi-log plot of the in-run data in 1 M NaCl.

The desorption behavior of the adsorbed DNA layer on the mica substrate was observed by adding monovalent salt into the gap buffer, which contained only divalent cations after the DNA deposition step. Once the DNA layer was adsorbed on the mica substrate in the MgCl_2_ solution, the gap buffer was replaced with buffer solutions containing different concentrations of NaCl. The interaction did not change obviously in the 10 mM NaCl (data not shown). However, when the gap buffer was changed to 100 mM NaCl, the hardwall decreased to less than 2 nm (data not shown). With a continuous increase in NaCl concentration to 1 M, we observed a lower hardwall of 1.3 ± 0.2 nm and long-range repulsion starting at a distance of approximately 60 nm, as shown in Figure 2. If the measurement error is taken into account, the hardwall decrease indicated desorption of the DNA layer on the mica surface beginning in 100 mM NaCl. The long-range repulsion measured in 1 M NaCl was likely due to the steric repulsion induced by the released DNA molecules suspended in the buffer solution. Thus, we can describe the DNA desorption kinetics to be the following: the DNA binding force becomes weaker in 100 mM NaCl, and the polymer molecules desorb completely from the mica substrate in 1 M NaCl solution.

According to the Poisson-Boltzmann equation, the density of divalent cations on a mica surface decreases dramatically if the divalent salts in bulk solution are replaced with monovalent salts, which directly results in the weakened binding strength of DNA molecules^11^. In this situation, the loosely attached DNA layer on the mica surfaces could be easily squeezed out under the action of the external normal load. The desorption of the DNA layer in 1 M NaCl solution was attributed to the decreased ion-ion correlation force due to the sharp reduction in the divalent ion concentration near the mica surfaces. Furthermore, the reduction of the divalent ion concentration also decreases the intramolecular forces in the DNA molecule itself. With the NaCl concentrations increasing from 10 mM to 100 mM and 1 M, the long-range repulsion force appears and the interaction range increases with NaCl concentration. This implies that the coiled DNA molecules unfold when monovalent ions with high concentration are introduced. A previous study also reported a similar change in intramolecular force for DNA molecules in different salt solutions^41^.

Additionally, we found that the concentration of monovalent cations necessary to loosen the attachment of the DNA layer was 10-fold higher that of the divalent cations, which is less than previously reported^15^. The selection of divalent ions to help DNA adsorption on a substrate also produced interesting results. Divalent transition metals, such as Ni^2+^ or Co^2+^ rather than Mg^2+^, were previously employed to protect DNA from desorption caused by monovalent cations. These cations have a better affinity with mica compared to Mg^2+^, which is effective for enhancing DNA adsorption^11^. Our results demonstrate that even Mg^2+^ alone can adsorb DNA well enough to prevent desorption.

### The adhesion energy between DNA and a mica surface

**Figure 3:**
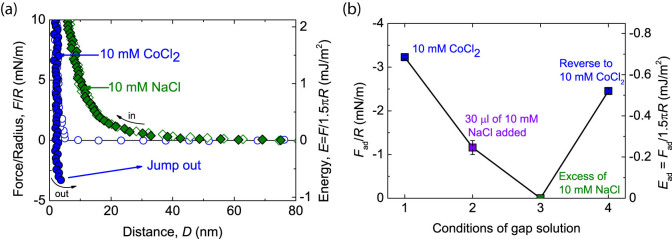
The correlation between the amount of added NaCl in the gap and the measured adhesion force between two mica surfaces. (a) After DNA adsorption in 10 mM CoCl_2_ (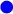), an equivalent amount of 10 mM NaCl was added into the surface gap. Then, the gap buffer was completely replaced with 10 mM NaCl (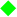). (b) The measured adhesion force decreased with an increase in the amount of 10 mM NaCl in a buffer solution, followed by a recovery to ¾ of the original magnitude when the buffer is returned to 10 mM CoCl_2_.

As described above, we measured the attraction force in 10 mM CoCl_2_ solution when two surfaces were separated. Due to the asymmetrical surface configuration (shown in Figure 1(b)), this measured adhesion of −3.2 ± 0.1 mN/m (*E*_ad_ approximately −0.67 mJ/m^2^) occurs at the interface between the DNA monolayer and mica, which reveals the binding energy of the DNA layer on mica. After several force-runs in CoCl_2_ solution, an equivalent amount of 10 mM NaCl buffer was injected into the gap buffer. The mixing of both monovalent and divalent cations resulted in the decrease of adhesion to −1.2 ± 0.2 mN/m. Following the complete replacement of the gap buffer with 10 mM NaCl solution, the adhesion no longer existed, and instead, long-range of electrostatic repulsion was observed starting at approximately 40 nm, as shown in Figure 3(a). At the end of the experiment, the gap buffer was returned to 10 mM CoCl_2_ solution, and the adhesion recovered to −2.5 ± 0.1 mN/m. The correlation between the addition of NaCl and the measured adhesion magnitude is shown in Figure 3(b). This phenomenon implies the crucial contribution of cobalt ions in a bulk environment to the binding energy between exposed DNA molecules in the adsorbed monolayer and the opposite mica surface.

**Figure 4:**
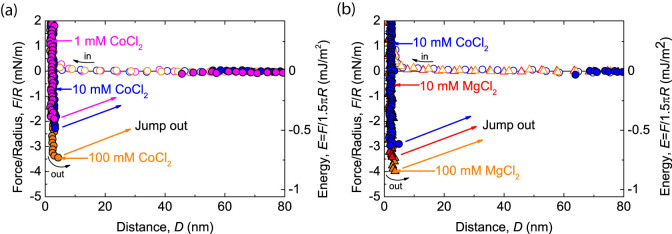
Effects of concentrations of divalent cations on the measured adhesion force when the DNA was initially incubated on a mica surface in a buffer solution containing CoCl_2_. (a) After DNA adsorption in 1 mM CoCl_2_ (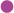), the concentration of the CoCl_2_ in buffer solution was increased to 10 mM (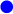) and 100 mM (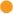). (b) After DNA adsorption in 10 mM CoCl_2_ (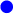), the buffer solution was replaced by 10 mM MgCl_2_ (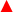) and 100 mM MgCl_2_ (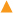).

To further explore this attractive interaction, we performed the measurements with different concentrations of cobalt and magnesium ions, as shown in Figure 4. In both experiments, the DNA was initially incubated on a mica substrate in CoCl_2_ solution, and then, the gap buffer was replaced with certain cobalt or magnesium solutions.

By changing the buffer solution, we found that once the DNA molecules were adsorbed on a mica surface, the adhesion increased with higher divalent ion concentrations regardless of cation species. Similar correlations have been found in increasing the concentrations of divalent cations to result in the enhancement of DNA adsorption strength on mica^42^. Moreover, as the proposed binding force, the counterion correlation was shown in a theoretical study to be enhanced with a higher fractional divalent surface density of DNA at a low ionic strength (I < 0.1 M)^11^, while the bound number of Co^2+^ ions on *λ* - DNA were found to increase linearly with Co^2+^ concentration up to 24 mM and then was constant^39^. From our SFA results, we consider that higher divalent concentration might have the potential to increase the adsorption efficiency of DNA on a mica substrate. However, there is also the possibility of DNA condensation or aggregation in a bulk solution at a high divalent concentration^39^,^43^, which may result in inefficient adsorption. In addition, no adhesion was measured when DNA was incubated in MgCl_2_ solution (Figure 1(a)) due to the strong steric repulsion resulting from the stiff DNA conformation. In addition, as mentioned above, the pretreatment of surface with Mg^2+^ induces relatively weaker binding compared to pretreatment with Co^2+^ and other transitional metal cations. We therefore conclude that both distributions of the transitional metal cations on the substrate interface and in the bulk liquid are of importance to the ion-correlation binding force.

## Conclusions

In summary, we experimentally studied the adsorption properties of λ-DNA on mica substrate using the SFA technique. DNA molecules were observed to form a moderately dense monolayer on mica substrate in the buffer solutions containing divalent ions of either Mg^2+^ or Co^2+^; however, the DNA exhibited different conformational structures for each of the two cations. By measuring the force-distance profiles between the DNA layer and mica, we characterized the DNA molecules that in MgCl_2_ solution were condensed wormlike coils with partial adsorption on the substrate and free loops or tails in the solution but in CoCl_2_ solution were smoothly lying flat in a 2D configuration on the substrate. In comparison, DNA molecules cannot adsorb on the mica substrate with a medium of monovalent cations, even with the addition of divalent salt. Furthermore, we measured an attraction between the DNA layer and mica of approximately −0.67 mJ/m^2^ in 10 mM CoCl_2_ solution, which is a direct measurement of the attractive force bridging the negatively charged DNA and the mica surface. Changing the ionic strength of divalent cations or increasing the monovalent cation concentration in bulk solution can tune this binding energy of DNA on the mica surface. We also noted that further elevating the ionic strength of divalent cations to approximately 800 mM may conversely introduce the DNA desorption^15^. This implies that the cation concentrations are limited to a narrow range for tuning the binding energy between DNA and a solid substrate. Considering that the ionic strength is usually beyond 1 M for DNA-based research, further work is necessary to explore the DNA binding mechanism on a mica substrate more thoroughly.

## Author Contributions

Y.K., Q.T. and Y.C. designed the research. Y.K. and Q.T. performed SFA experiments. Y.K. analyzed the experimental data. Y.K., Q.T., G.W., W.S. and Y.C. contributed to results discussion. Y.K. and Y.C. wrote the paper.

## Supplementary Material

Supplementary Information Supplementary
