# Supplementary material for: Study of DNA adsorption on mica surfaces using a surface force apparatus
Source: Sci Rep. 2015 Feb 13;5:8442. doi: 10.1038/srep08442 (PMC4326959; doi:10.1038/srep08442)
Supplement: Supplementary Information — Supplementary [file srep08442-s1.pdf]

# Study of DNA adsorption on mica surfaces using a surface force apparatus

Yajing Kan, Qiyan Tan, Gensheng Wu, Wei Si, Yunfei Chen*

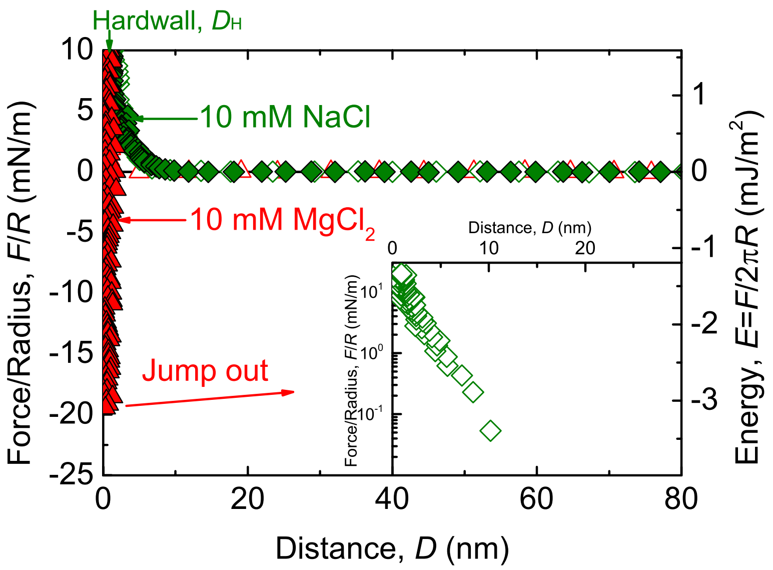


Figure S1. Force-distance profiles between DNA layer and mica when DNA was initially incubated on mica in the buffer solution containing 10 mM of NaCl. After DNA adsorption (◆), the buffer solution was replaced by 10 mM $MgCl_2$ (▲). Inset shows the semi-log plot of the in-run data in 10 mM NaCl.

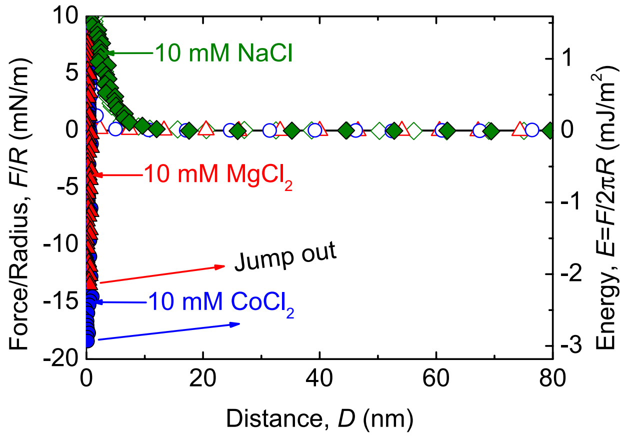


Figure S2. Force-distance profiles between two bare mica surfaces in 10 mM of NaCl (◆), $MgCl_2$ (▲) and $CoCl_2$ (●), respectively.
